# State or National Medical Service

**Published:** 1918-12-14

**Authors:** G. T. K. Maurice


### THE EDITOR'S LETTER-BOX.

[Correspondence on all subjects is invited, but we cannot in any way be responsible for the opinions expressed by our correspondents, who must give their name and address as a guarantee of good faith, but not necessarily for publication. Correspondents are reminded that brevity of style and conciseness of statement greatly facilitate early insertion.]

### State or National Medical Service.

*To the Editor of* The Hospital.

Sir,—You are the judge whether or no any comment is due from me on your correspondent whose contribution is on page 197 of The Hospital of December 7.

I was talking with others about panel practice, a thing all present thought a failure. One said that there would be State Medical Service, and another that none could devise one, and I that I had long thought much pointed that way, and that I did not think it was very difficult to design a Service, and that in many ways it would be advantageous alike to patients and doctors. Then one said, "You had better design one, then. Cut along and do it." He spoke scornfully. I took up the challenge, and wrote the article to which you gave the hospitality of your columns. This explains how I came to write.

I am a dispassionate observer of the trend of events, not personally interested in what may come, therefore the more likely to be judicial. My opinion is that the public will demand a State Medical Service. It is very likely the public will not have definite views about a State Medical Service, but that will not prevent the demand. The public demanded the panel system without knowing what it meant. I do not imply that the public formulated the organisation it got, but it asked for something of the kind. The medical profession did not set itself collectively to organise a system in answer to the demand, and so got pushed into a system made by men not representative of the medical profession. The medical profession then firmly opposed the new system, but, because it was not organised and had no alternative plans, was "stampeded," to use a word in the article "Need for Organisation," which appears in this week's Hospital, page 192, into accepting a system which is not pleasing either to the profession or the public. I think that again a vague demand from the public is coming, that the necessities of a Minister of Health will give the demand concrete shape, whether the medical profession assists in the shaping or no, and that that shape may be forced on the profession whether they like it or no. I therefore think the medical profession will be wise to prepare schemes for organisations of such nature that some one of them shall be acceptable to it and the public, or less objectionable to it than a scheme made by politicians, whilst the medical profession, unorganised and unready, is crying out, "State service is not possible. We won't accept it." My scheme is only one possible scheme. I hope to see others in the field. Many think a State Medical Service neither necessary nor desirable. They may be right. It is very desirable that those who so think should set forth fully the reasons for their opinion and array the arguments for and against. To do so will be true statesmanship, and if the weight of argument against is greater than the weight of argument for State Medical Service, it may prevent an ill-considered demand and a costly mistake. I have no doubt you, sir, will as readily extend the hospitality of your columns to such arguments as you did to my article.—Yours faithfully,

G. T. K. Maurice.

December 6, 1918.

[*** Our columns are open to all views, and we should welcome correspondence on the subject.—Ed. The Hospital.]

### The Right to Send Private Patients to District Hospitals.

*To the Editor of* The Hospital.

